# Detection of *Brucella* spp. in raw milk from various livestock species raised under pastoral production systems in Isiolo and Marsabit Counties, northern Kenya

**DOI:** 10.1007/s11250-020-02389-1

**Published:** 2020-09-18

**Authors:** Martin Wainaina, Gabriel O. Aboge, Isaac Omwenga, Catherine Ngaywa, Nicholas Ngwili, Henry Kiara, George Wamwere-Njoroge, Bernard Bett

**Affiliations:** 1 International Livestock Research Institute, Nairobi, Kenya; 2 Department of Public Health Pharmacology and Toxicology, College of Agriculture and Veterinary Sciences, University of Nairobi, Nairobi, Kenya; 3 Centre for Biotechnology and Bioinformatics, College of Biological and Physical Sciences, University of Nairobi, Nairobi, Kenya

**Keywords:** *Brucella*, Milk, ELISA, PCR, Pastoral

## Abstract

**Introduction:**

Brucellosis is an important zoonotic disease in Kenya, and identifying the bacteria in milk is important in assessing the risk of exposure in people.

**Methods:**

A cross-sectional study that involved 175 households was implemented in the pastoral counties of Marsabit and Isiolo in Kenya. Pooled milk samples (n = 164) were collected at the household level, and another 372 were collected from domesticated lactating animals (312 goats, 7 sheep, 50 cattle and 3 camels). Real-time polymerase chain reaction (qPCR) testing of the milk samples was performed to identify Brucella species. Brucella anti-LPS IgG antibodies were also detected in bovine milk samples using an indirect enzyme-linked immunosorbent assay (ELISA).

**Results:**

Based on the qPCR, the prevalence of the pathogen at the animal level (considering samples from individual animals) was 2.4% (95% confidence interval (CI) 1.1–4.5) and 3.0% (CI: 1.0–7.0) in pooled samples. All 14 samples found positive by qPCR were from goats, with 10 contaminated with B. abortus and 4 with B. melitensis. The Brucella spp. antibody prevalence in bovine milk using the milk ELISA was 26.0% (95% CI: 14.6–40.3) in individual animal samples and 46.3% (95% CI: 30.7–62.6) in pooled samples.

**Conclusion:**

The study is the first in Kenya to test for Brucella spp. directly from milk using qPCR without culturing for the bacteria. It also detected B. abortus in goats, suggesting transmission of brucellosis between cattle and goats. The high prevalence of Brucella spp. is a significant public health risk, and there is a need for intervention strategies necessary in the study area.

**Electronic supplementary material:**

The online version of this article (10.1007/s11250-020-02389-1) contains supplementary material, which is available to authorized users.

## Introduction

Brucellosis is an important zoonotic disease caused by some bacteria in the genus *Brucella*. Of the 12 identified species within the genus, three (*B. melitensis*, *B. abortus* and *B. suis*) are the ones mostly associated with brucellosis in humans and certain animals (Al Dahouk et al. 2017). The disease impoverishes many through its negative impacts on livestock production and trade as well as diminished productivity in people.

The incidence rate of human brucellosis in Kenya is currently not estimated (Njeru et al. 2016). It is the fourth most important zoonosis in the country (Munyua et al. 2016) and is notifiable when it occurs in animals. Most cases are reported in the arid and semi-arid land (ASAL) areas where pastoralism is the main source of income (Njeru et al. 2016). It is likely to be endemic in these areas because livestock are often raised in large herds, are communally herded and bred and interact with wildlife (such as buffaloes) which may act as reservoirs for some *Brucella* species (McDermott and Arimi 2002). Humans get exposed to *Brucella* spp. through consumption of contaminated raw or poorly cooked animal source foods (ASF), contact with infected tissues such as aborted foetuses and taking care of sick animals (Corbel 2006). Consumption of raw or undercooked milk is an important way through which many people get exposed. This includes women and children who do not participate in some risky livestock husbandry practices such as herding and slaughtering (Corbel 2006; Roesel and Grace 2014).

Identifying this hazard in milk is important in assessing the food safety risk posed to the public. We therefore implemented this study to detect the bacteria using molecular tools in milk collected from individual lactating animals and milk pooled by household members, with an aim of estimating the prevalence of the bacteria in the milk as well as determining species of the bacteria involved.

## Materials and methods

**Fig. 1:**
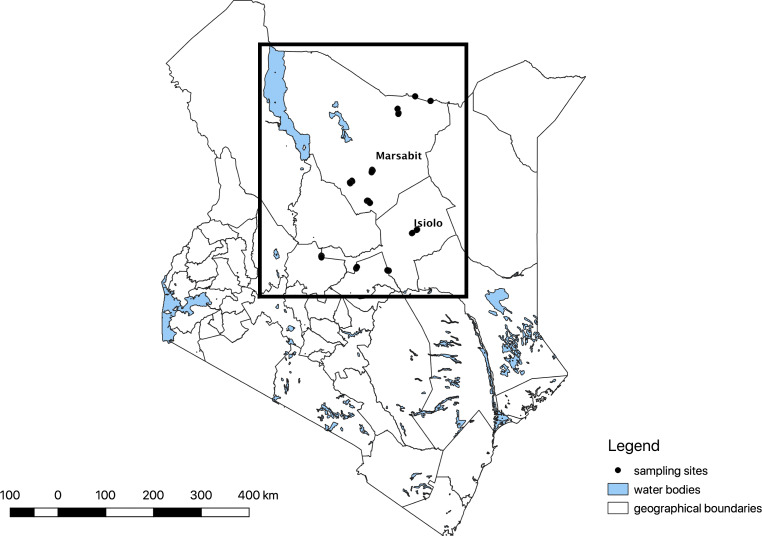
Marsabit and Isiolo Counties shown in Kenya with the locations of households sampled highlighted in black dots. The study was implemented between June 2016 and February 2017

The study was part of a larger study investigating milk-borne pathogens in pastoral Kenya as previously described by Omwenga et al. 2019 and Ngaywa et al. 2019. It was implemented in Burat, Kinna, Merti and Oldonyiro Wards in Isiolo County and Karare, Korr, Laisamis, Moyale, Sololo and Turbi Wards in Marsabit County. Isiolo and Marsabit Counties are part of the ASAL areas of Kenya (Fig. 1) and are among the most resource-scarce counties in Kenya.

### Study design

A cross-sectional study design was used, and households as well as individual animals were the basic units of sampling. Pooled milk and that from three randomly selected lactating animals were collected. A household in this case referred to a home with a family and livestock of interest (bovines, small ruminants or camels) with at least three lactating animals and belonging to a village in either of the two study counties. The number of households to use was determined using a multistage sampling formula (Dohoo et al. 2003). Previous studies in Marsabit estimated the individual cattle seroprevalence of *Brucella* spp. at 13.5% (Osoro et al. 2015). Given that there were no reliable estimates of brucellosis prevalence in milk in the area, we assumed a 50% prevalence, 95% desired level of confidence and 10% desired precision. This provided a sample size of 97.

We assumed that households in a given area (e.g. village/grazing camps in our case) shared similar characteristics. Hence, chances of milk contamination from these spatial units would tend to be similar. Adjusting for clustering on the sample size was done using the formula *n*’ = *n*(1 + *ρ*(*m*–1)), where *n*’ is the new sample size, *n* is the original sample size estimate, *ρ* is the intracluster correlation coefficient and *m* the number of animals to be sampled in each cluster (Dohoo et al. 2003). We assumed a target household number of 20 per village and an intracluster correlation coefficient of 0.03. This provided a design effect of 1.57 and a final sample size of 152 households.

To select households, 10 wards were first conveniently selected based on closeness to road networks across the two areas. Villages were randomly selected from a sampling frame of each ward and four villages were targeted within each ward. Lastly, 15 to 17 households were assigned to each village. Within each village, systematic sampling along transects marked by the road networks was used to randomly recruit households. Every fifth household was recruited if it had livestock of interest (bovines, small ruminants or camels). Those that failed to meet this criterion were replaced by their immediate neighbours.

### Sample and data collection

For every livestock species of interest kept in a selected household, up to three lactating animals (per species) were sampled by experienced veterinary technicians and kept as independent samples. One pooled sample was also obtained from the milk pooled by household members at the household level. Households in the study area generally pooled milk by species, and pooled milk samples typically represented milk from a single species. Households owned variable numbers of lactating animals, and therefore, the number of animals contributing to a pool was not determined. Individual milk samples did not constitute pooled milk samples. Pooled milk was kept for home consumption only.

Milk from individual animals was collected after fore-stripping from all the teats of the lactating animal after disinfection using sterile swabs containing 70% ethyl alcohol solution. Around 10 ml of the sample was collected using sterile 50-ml Falcon tubes. For household pooled milk, the milk stored at the household for consumption at the time of sampling was obtained from the storage container into the sterile Falcon tube.

Falcon tubes were identified using unique barcode labels. Each sample was later aliquoted and the aliquots for this work treated using 0.02% sodium azide for preservation before freezing and transportation to the International Livestock Research Institute (ILRI), Nairobi, for analysis and storage. Aliquots were also identified using barcodes, and these were linked to the Falcon tube barcode identifiers. An aliquot database was maintained on MS Excel software. Data on animal and household characteristics were collected using electronic forms administered using the Open Data Kit (ODK) mobile application (https://opendatakit.org/). Data collection happened between June 2016 and February 2017.

### Laboratory analysis

*Brucella* deoxyribonucleic acid (DNA) and antibodies in raw milk of lactating animals were detected by real-time polymerase chain reaction (qPCR) and enzyme linked immunosorbent assay (ELISA), respectively.

### DNA extraction and qPCR testing

Aliquots of 1.5 ml of each sample were spun at 5000 g for 20 min to separate the cream from the milk plasma. Two hundred microlitres (200 μl) of the milk sediment was collected and pipetted into Eppendorf tubes for DNA isolation using the DNeasy® Blood & Tissue Kit (Qiagen, Hilden, Germany) as described in the slightly modified procedure in bullet 1 in the supplementary material.

qPCR testing was done using the primers and TaqMan probes previously described by Probert et al. (2004) and the assay was used in singleplex. All the milk samples were analysed in duplicates for *Brucella* spp. by targeting the bcsp31 gene. All the positive samples were further tested in duplicates using both the *B. abortus* and *B. melitensis* primers and TaqMan probes targeting insertions of an IS711 element downstream of the alkB gene and BMEI1162, respectively. Details of qPCR testing are clarified in bullet 2 in the supplementary material. Some qPCR positives were sequenced for confirmation and one sequence was uploaded to GenBank (Accession number MK531856).

### Milk-ELISA testing

Bovine milk samples were tested for anti-lipopolysaccharide (LPS) *Brucella* spp. antibodies using the commercially available PrioCHECK® *Brucella* Ab 2.0 indirect ELISA kit (Prionics AG, Schlieren-Zurich, Switzerland). One hundred microlitres (100 μl) of the milk plasma was used to test after the milk aliquots were spun at 5000 g for 15 min. Analysis was done according to the manufacturer’s instructions accompanying the kit. Percent positivity (PP) was calculated as per the manufacturer’s instructions, and those with PP below 25% were regarded as negatives while those above as positives. Laboratory results were recorded and calculated using MS Excel software.

### Data management and analysis

ODK forms were downloaded from the ILRI servers and saved as comma delimited files (.csv). The data were cleaned and merged on R statistical environment version 3.4.1 before the merged document was imported into STATA version 13 for analysis. Descriptive analyses were done to determine the prevalence of *Brucella* spp. based on the PCR and ELISA tests. Fisher’s exact tests were also performed to determine whether the prevalence values varied by county as well as sample type.

## Results

A total of 171 households were sampled in the survey, with 95 households being from Marsabit County and the rest from Isiolo. A large proportion (90.5%) of the households sampled kept goats and 81.5% kept sheep. Cattle were kept by 60.1% of the households, and 88.6% of these households kept them with (either) sheep and (/or) goats. A lower proportion of households (34.8% and 17.4%) kept camels and chickens, respectively. A total of 536 milk samples were available for laboratory analyses. Of these, 267 were collected from Marsabit County while 269 from Isiolo County (Table 1). There were 372 samples from individual animals (312 from goats, 7 from sheep, 50 from cattle and 3 from camels) and 164 pooled milk samples.

**Table 1:** Distribution of the milk samples screened for *Brucella* spp. using real-time PCR

Milk sample	Livestock species	Number sampled	Number positive	% Prevalence (95% CI)
Individual	Goats	312	9	2.9 (1.3–5.4)
	Sheep	7	0	0.0 (0–41.0)
	Cattle	50	0	0.0 (0–7.1)
	Camels	3	0	0.0 (0–70.8)
Pooled	Goats	97	5	5.2 (1.7–11.6)
	Sheep	1	0	0.0 (0–97.5)
	Cattle	41	0	0.0 (0–8.6)
	Camels	11	0	0.0 (0–28.5)
	Sheep and goats	5	0	0.0 (0–52.2)
	Mixed species	9	0	0.0 (0–33.6)
All		536	14	2.6 (1.4–4.3)

### Molecular detection

**Fig. 2:**
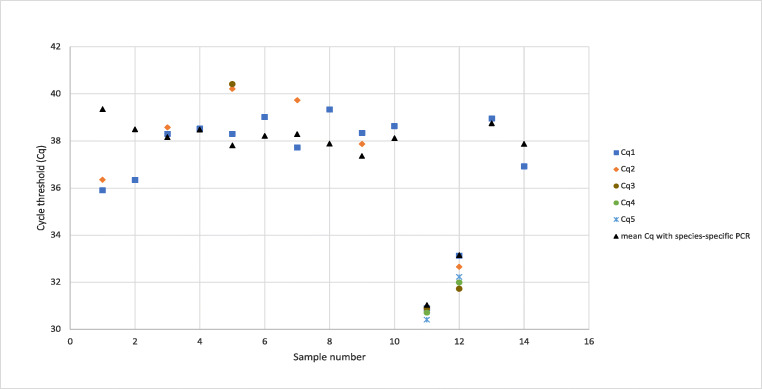
Cq values of all positive samples by real-time PCR, with their repeats and mean Cq values shown in different colours. Samples were analysed between two to five times. Samples 1, 2, 11 and 12 were detected as *B. melitensis* with the rest being *B. abortus*. Samples 1 and 12 were pooled samples, with the rest being individual ones

A total of 536 milk samples were available for laboratory analyses. *Brucella* spp. was detected in 14 milk samples, with 10 being identified as *B. abortus* and the remaining 4 as *B. melitensis*. The types and numbers of livestock sampled and the prevalence of *Brucella* spp. based on real-time PCR and by sample type are given in Table 1. A summary of the Cq values for the positives can be found in Fig. 2.

#### Prevalence in individual animal samples

Based on the real-time PCR results, all contaminated milk samples were obtained from goats (Table 1). The overall prevalence in individual samples was 2.4% (95% confidence intervals (CI): 1.1–4.5). The prevalences observed from individual animals sampled in Isiolo and Marsabit Counties were 3.9% (95% CI: 1.7–7.6) and 0.6% (95% CI: 0.0–3.3), respectively. A significant difference was observed in the prevalence of the bacteria between the two counties (*p* = 0.045).

A summary of the distribution of the PCR positives with the wards is given in Table 2.

**Table 2:** The relationship between the sampling wards and sample types for the positives found in the study using the two diagnostic tests used

		PCR						ELISA					
County	Ward	Individual			Pool			Individual			Pool		
		Positive	Total	%	Positive	Total	%	Positive	Total	%	Positive	Total	%
Marsabit	Karare	0	11	0.0	0	11	0.0	1	3	33.3	4	10	40.0
	Korr	0	49	0.0	0	20	0.0						
	Laisamis	0	38	0.0	0	20	0.0						
	Moyale	1	25	4.0	1	20	5.0	0	2	0.0	3	11	27.3
	Sololo	0	12	0.0	0	10	0.0	0	3	0.0	0	4	0.0
	Turbi	0	33	0.0	0	18	0.0				3	4	75.0
Isiolo	Burat	5	39	12.8	2	13	15.4	0	2	0.0	0	1	0.0
	Kinna	1	57	1.8	1	18	5.6	11	36	30.6	8	10	80.0
	Merti	1	58	1.7	1	17	5.9	1	3	33.3	1	1	100.0
	Oldonyiro	1	50	2.0	0	17	0.0	0	1	0.0			
Total		9	372	2.4	5	164	3.0	13	50	26.0	19	41	46.3

#### Prevalence in pooled samples

The overall prevalence observed by PCR from pooled milk samples was 3.0% (95% CI: 1.0–6.9), with 1.0% (95% CI: 0.0–5.5) and 6.2% (95% CI: 1.7–15.1) being observed in Marsabit and Isiolo Counties, respectively. There was no statistically significant difference observed between the two counties (*p* = 0.081).

### *Brucella* antibody-prevalence

A total of 91 samples, 37 from Marsabit and 54 from Isiolo, were screened using milk ELISA test. None of the ELISA positives was PCR positive.

#### Prevalence in individual animal samples

Fifty samples were collected from individual cattle for ELISA testing. The overall antibody prevalence was 26.0% (95% CI: 14.6–40.3). Antibody prevalence in Marsabit and Isiolo Counties were 12.5% (95% CI: 0.3–52.7) and 28.6% (95% CI: 15.7–44.6), respectively. There was no significant difference observed in the prevalences by county (*p* = 0.662). The distribution of ELISA positives between sampled wards can be found in Table 2.

#### Prevalence in pooled samples

Forty-one samples were collected from cattle for ELISA testing. The overall prevalence observed was 46.3% (95% CI: 30.7–62.6). Antibody prevalence in Marsabit and Isiolo Counties were 34.5% (95% CI: 17.9–54.3) and 75.0% (95% CI: 42.8–94.5), respectively. A statistically significant difference in the prevalences between the counties was observed (*p* = 0.037).

#### Sample type comparisons

There was no significant difference observed between PCR results from individual and pooled samples (*p* = 0.77). However, ELISA results showed statistically significant differences when individual and pooled samples results were compared (*p* = 0.05).

## Discussion

This study determined the prevalence of *Brucella* spp. in pooled and individual lactating animal milk samples that were collected in Marsabit and Isiolo Counties of Kenya. Two critical results generated include (i) determination of the prevalence of *Brucella* spp. in milk and (ii) identification of *Brucella* spp. that are prevalent in lactating animals in the study areas. This information is critical for designing brucellosis interventions in the area.

While it is known that *B. abortus* has cattle as its primary natural host, infection in small ruminants has been demonstrated before (Ocholi et al. 2004). Transmission of other *Brucella* species to atypical hosts has also been observed in different parts of the world (Díaz Aparicio 2013; Menshawy et al. 2014; Muendo et al. 2012; Wareth et al. 2015). These findings support the evidence that keeping cattle together with small ruminants could be a risk factor for the spread of animal brucellosis in the country (Muendo et al. 2012). A large proportion (88.6%) of our study households kept cattle with (either) sheep and (/or) goats. About 90% of the households involved in our study kept goats and all of the PCR positives were goats, suggesting the importance of goats in transmission of brucellosis. Other authors have also highlighted the likely importance of goats in transmission of brucellosis in the country and region (Osoro et al. 2015; Viana et al. 2016). Our findings help better understand the epidemiology of the disease in animal populations. To the best of the authors’ knowledge, this is the first time *B. abortus* has been detected in goats in Kenya.

*Brucella* DNA detection in milk using qPCR may be hampered when its presence is intermittent, short-lived or the amount is too low for detection. This, compounded by the inhibition of the PCR reaction, could lead to false negatives, especially in DNA extracted from milk due to the presence of residual milk proteins or calcium ions in the eluted DNA. The inclusion of inhibition controls is therefore advised, as was done in our study (Capparelli et al. 2009; Schrader et al. 2012; Wilson 1997). The use of PCR in detecting the pathogen may not mean the identified pathogen is viable but can give a good indication of the risk in the food without the need for culturing for the pathogen under high biocontainment. This is especially important for Kenya and other low- and middle-income countries which often lack safe facilities to culture for the bacteria.

The study showed considerable PCR prevalence in Kinna and Burat Wards of Isiolo County. These areas are near high populations of wildlife, and it is possible that intense livestock-wildlife interactions may be responsible for increased transmission of the bacteria. Other places in the country with increased interactions with wildlife have been shown to have higher livestock brucellosis seroprevalence (Enström et al. 2017).

There were no significant differences observed in the prevalence of the pathogen by PCR with the sample type. Pooled milk however had a significantly higher antibody-prevalence when compared with that of individual samples. Bulking of milk meant for consumption in markets from different sources is thought to have a dilution effect to the pathogen (Hoffman et al. 2016) which can reduce sensitivity of PCR. Pooling at the household level may however increase the chances of antibody-positivity of the batch of pooled milk when several antibody-positive animals contribute to the pool.

The detection of the bacteria in milk meant for human consumption, especially in these communities that are known to consume raw milk (Osoro et al. 2015; Kaindi et al. 2012), demonstrates a high risk of transmission of the bacteria. Studies have shown that ewes (Tittarelli et al. 2005), cattle (Lapraik and Moffat 1982), goats (Higgins et al. 2017) and camels (Wernery et al. 2007) that are infected with the bacteria can continue shedding the bacteria in milk for long periods of time. Some food-producing animals have been shown to be superspreaders of the bacteria (Capparelli et al. 2009). The low number of individuals observed to be PCR-positive in this study therefore is still dangerous as other animals in the herd and indeed households that draw milk from their infected herds on regular basis for subsistence can get continually exposed over the length of time the animal is shedding the bacteria. The presence of an infected animal in a herd has been shown to increase the odds of human exposure 3 to 6 times in other parts of the country (Kairu-Wanyoike et al. 2019; Osoro et al. 2015). The villages sampled also fell along the major highways, and at times supply milk to the local market centres due to the convenience of the road network. When this happens, it is mostly sold to local traders, who then supply major traders in the local markets. In this regard, there is the likelihood that infected milk can get into the milk value chain. Drinking milk that is boiled leads to killing of the bacteria and should therefore be encouraged. Ingestion of raw milk or mixing of boiled milk with fresh milk batches may also lead to contamination of the milk with the bacteria and pose risk of infection (Daniel and Cornelius 2015).

Other lower *Brucella* spp. prevalence figures in cattle from other ASAL regions in Kenya have been published (Kadohira et al. 1997; Osoro et al. 2015; Kairu-Wanyoike et al. 2019). These studies however tested serum samples and used heterogeneous livestock populations and not lactating animals only as was done in this study. A study done by Chota et al. (2016) in West Pokot, an ASAL area as well, reported milk antibody positivities of 21.9% and 21.2% in the years 2012 and 2014 using the milk ring test. Other lower values have been reported in other parts of Kenya with different climatic conditions (Kang’ethe et al. 2004; Kang’ethe et al. 2007). Our study demonstrated considerable levels of exposure to *Brucella* spp. in lactating herds, a finding that shows the public health risk posed in the study areas.

Our study had several limitations. Given the relatively small sample sizes of sheep and camels, and to a lesser extent, cattle, which are important livestock in these areas, our study lacked sufficient power to determine prevalence with the desired precision in these species. This is evidenced by the wide confidence intervals for sheep and camel prevalence figures. We recommend future studies with sufficient statistical power in investigating these species as they could also play an important role in human brucellosis in the area. The use of the commercially available indirect ELISA tests in screening the cattle milk samples could have been influenced by cross-reactivity with antibodies from other gram-negative bacteria (Corbel 2006), or even suffered from cut-offs that are not appropriate for milk samples from the region. Due to the lack of commercially available *Brucella* spp. milk antibody tests such as ELISAs that are developed and validated for goats, sheep and camels (J. Voss, personal communication, 2016), milk from these animals was not tested. Testing the exposure of herds of all the different species would have been of great value in understanding the food safety risk in the study areas. There also exists a need of developing commercially available antibody-testing kits with high sensitivity and specificity for testing brucellosis using milk in these species. While the ICC estimates used in our study are considerably lower than what has been estimated for *Brucella* in this setting in Kenya (Kairu-Wanyoike et al. 2019), the proportion of positive cases found was largely below 50% and therefore likely to have little impact on the interpretation of our results.

In conclusion, prevalence of exposure (based on ELISA) and infection (based on PCR) of brucellosis was determined in this study from lactating animals in pastoral parts of Kenya. The study was the first in the country to have identified the hazard associated with milk directly using PCR and the second to identify *Brucella* spp. in atypical hosts after work by Muendo et al. (2012). Kenya has no published brucellosis control strategy (Njeru et al. 2016). Human brucellosis has however been shown to be high in the study areas (Osoro et al. 2015; Njeru et al. 2016). Measures that can be used to limit human exposure include boiling milk and culling of infected animals. Even though not routinely performed in Kenya, vaccination control strategies that target goats may also be important in controlling spread of the disease to humans in the study area. While beneficial in many other settings, pasteurization in Kenya has been shown to have the counterproductive effect of increasing the price of market milk beyond the reach of many consumers of a low socio-economic status, thereby limiting access to the nutritious food (Grace 2017). We recommend the need to sensitize the local communities in the area of the risk of exposure to the pathogen via household milk consumption and the importance of boiling milk from all species before consumption at the household. Our study has generated additional evidence on the distribution of *Brucella* spp. in milk and demonstrated the need to apply molecular diagnostic tools to characterize the pathogen.

## Electronic supplementary material

ESM 1 (DOCX 24 kb)
