# Supplementary material for: Detection of Brucella spp. in raw milk from various livestock species raised under pastoral production systems in Isiolo and Marsabit Counties, northern Kenya
Source: Trop Anim Health Prod. 2020 Sep 18;52(6):3537–44. doi: 10.1007/s11250-020-02389-1 (PMC7606284; doi:10.1007/s11250-020-02389-1)
Supplement: Supplementary file 1 — (DOCX 24 kb) [file 11250_2020_2389_MOESM1_ESM.docx]

**Supplementary material:**

**Detection of *Brucella* spp. in raw milk from various livestock species raised under pastoral production systems in Isiolo and Marsabit Counties, northern Kenya**

Martin Wainaina^a^*, Gabriel O. Aboge^b, c^, Isaac Omwenga^a,b^, Catherine Ngaywa^a,c^, Nicholas Ngwili^a^, Henry Kiara^a^, George Wamwere-Njoroge^a^, Bernard Bett^a^

1. *International Livestock Research Institute, Nairobi, Kenya.*
2. *Department of Public Health Pharmacology and Toxicology, Faculty of Veterinary Medicine, University of Nairobi, Nairobi, Kenya.*
3. *Centre for Biotechnology and Bioinformatics, College of Biological and Physical Sciences, University of Nairobi, Nairobi, Kenya.*

* Corresponding author, email: [m.wainaina@cgiar.org](mailto:m.wainaina@cgiar.org)

**1. DNA extraction**

Aliquots of 1.5ml of each milk sample were spun at 5000g for 20 minutes to separate the cream. Two hundred microlitres (200 µl) of the milk sediment was collected and pipetted into labelled Eppendorf tubes for DNA isolation using the DNeasy® Blood & Tissue Kit (Qiagen, Hilden, Germany). A negative extraction control (NEC) was included in every extraction batch and this included all the extraction reagents and apparatus without any sample being used. Proteinase K (30 µl) and 300µl of Buffer AL (lysis buffer) were added into labelled tubes, vortexed and incubated at 56˚C for 1 hour for proper lysis. After discarding the flow-through, DNA was precipitated by adding 200µl of absolute ethanol and later centrifuging at 8000g for 1 minute. The precipitated DNA was washed with 500μl of buffer AW1 twice by centrifuging at 8000g for 1 minute. This first wash was done using the same collection tubes with blot drying done in between the two washes.

Similarly, washing with 500μl of buffer AW2 was performed twice by centrifuging at 20000g for 3 minutes. Blot drying was done between the two washes with buffer AW2. A dry spin was done at the end to remove residual wash buffers before elution using 100μl of buffer AE (elution buffer). The tubes were incubated for 1 hour at room temperature before being spun at 6000g for 1 minute. The concentration and purity of the eluted DNA was determined using the NanoDrop™ 2000 spectrophotometer.

**2. Realtime PCR analysis**

A singleplex real-time PCR was done using the primers and TaqMan probes previously described by Probert et al., (2004). The primers and probes were purchased Macrogen Inc. (Amsterdam, Netherlands) and are outlined in Table 1 below.

All the extracted DNA (and the NECs from the extraction process) were analyzed in duplicates for *Brucella* spp. by targeting the bcsp31 gene. All the positive samples were further tested in duplicates using both the *B. abortus* and *B. melitensis* primers and TaqMan probes targeting the alkB gene and BMEI1162 respectively. An inhibition control which consisted of one part extracted DNA and one part of positive control was included in every qPCR run as well. The 25μl reaction mixture for all the assays contained 1X PerfeCTa FastMix II (Quantabio, Beverly, MA, USA), 400nM each of forward and reverse primers, 200nM of TaqMan probe, 2μl of DNA template and nuclease free water. A positive control (Bruce ladder Suis, Ingenasa, Madrid) was used for the *Brucella* spp. assay. The LightCycler® Nano (Roche, Basel, Switzerland) was used to analyze the samples and interpretation was done by the LightCycler® Nano software version 1.0.7 (Roche, Basel, Switzerland). Absolute quantification and automatic quantification were the conditions set on the software for quantification threshold (Cq) calling and values with average Cq values higher than 40 when tested at least twice were not regarded as positives, unless they were also detected by one of the species-specific assays (see Figure 2 in main text).

Purification of amplification products was done on some positive samples from the genus-specific assay using the QIAquick Gel Extraction Kit (Qiagen, Hilden, Germany) for subsequent Sanger sequencing at Macrogen Inc. (Amsterdam, Netherlands). Sanger sequences were analysed using the CLC Main Workbench 7.8.1 software package (CLC bio, Aarhus, Denmark) and the consensus sequences obtained were subjected to BLASTn searches (<https://blast.ncbi.nlm.nih.gov/Blast.cgi>) to confirm *Brucella* genus detected by the qPCR assay. One sequence from the genus-specific assay was uploaded to the GenBank database (<https://www.ncbi.nlm.nih.gov/genbank/>) and assigned accession number **MK531856**.

| **Target** | **Primer/Probe name** | **Sequence and modifications (5' → 3')** |
| --- | --- | --- |
| *Brucella* spp. bcsp31 gene | Forward | GCTCGGTTGCCAATATCAATGC |
|  | Reverse | GGGTAAAGCGTCGCCAGAAG |
|  | Probe | 6-FAM/AAATCTTCCACCTTGCCCTTGCCATCA/BHQ_1 |
| *B. abortus* IS711 insertion element of alkB gene | Forward | GCGGCTTTTCTATCACGGTATTC |
|  | Reverse | CATGCGCTATGATCTGGTTACG |
|  | Probe | 5HEX/CGCTCATGCTCGCCAGACTTCAATG/3BHQ_2 |
| *B. melitensis* IS711 insertion element of BMEI1162 | Forward | AACAAGCGGCACCCCTAAAA |
|  | Reverse | CATGCGCTATGATCTGGTTACG |
|  | Probe | 5TexRd-XN/CAGGAGTGTTTCGGCTCAGAATAATCCACA/3BHQ |

Table 1: A summary of the primers and probes for use to detect *Brucella spp*. Primers and probes were purified by high performance liquid chromatography (HPLC) by the manufacturer before use.

**References**

Probert, W. S., Schrader, K. N., Khuong, N. Y., Bystrom, S. L., & Graves, M. H. (2004). Real-Time Multiplex PCR Assay for Detection of Brucella spp., *Journal of Clinical Microbiology*, *42*(3), 1290–1293. https://doi.org/10.1128/JCM.42.3.1290
